# Colonic J-pouch versus side-to-end anastomosis for rectal cancer: a systematic review and meta-analysis of randomized controlled trials

**DOI:** 10.1186/s12893-021-01313-0

**Published:** 2021-08-21

**Authors:** Zheng Wang

**Affiliations:** Department of Science and Technology, West China Hospital, Sichuan University, Chengdu, China

**Keywords:** Rectal cancer, Colonic J-pouch anastomosis, Side-to-end anastomosis, Meta-analysis

## Abstract

**Background:**

This study aims to compare colonic J-pouch and side-to-end anastomosis for rectal cancer in terms of surgical and bowel functional outcomes and quality of life (QoL).

**Methods:**

A systematic literature search was performed in PubMed, Embase and Cochrane. The last search was performed on March 28, 2021. All randomized controlled trials comparing colonic J-pouch with side-to-end anastomosis for rectal cancer were enrolled. The main outcomes were bowel functional outcomes and QoL. The secondary outcomes were surgical outcomes including operative time, postoperative hospital stay, complications, and mortality.

**Results:**

Nine articles incorporating 7 trials with a total of 696 patients (330 by J-pouch and 366 by side-to-end) were enrolled in this meta-analysis. The bowel functional outcomes were comparable between J-pouch and side-to-end groups in terms of stool frequency, urgency, and incomplete defecation at the short term (< 8 months), medium term (8–18 months), and long term (> 18 months) follow up evaluations. No difference was observed between groups with regards to QoL (SF-36: physical function, social function, and general health perception). Besides, surgical outcomes were also similar in two groups.

**Conclusion:**

The currently limited evidence suggests that colonic J-pouch and side-to-end anastomosis are comparable in terms of bowel functional outcomes, QoL, and surgical outcomes. Surgeons may choose either of the two techniques for anastomosis. A large sample randomized controlled study comparing colonic J-pouch and side-to-end anastomosis for rectal cancer is warranted.

## Background

Total mesorectal excision (TME) is the best available treatment for rectal cancer. With the advancement of surgical techniques, the majority of patients with mid and upper rectal cancer can undergo a sphincter-saving TME procedure. After TME, the most widely used reconstructive technique is straight coloanal anastomosis. However, because the sigmoid colon is usually excised during surgery which decreases the storage volume of stool, there is a common problem seriously influencing the life quality of patients, including increased tool frequency, urgency and incontinence, which is termed as “anterior resection syndrome (ARS)” [1]. About 19–56% of patients would suffer from ARS [2–6]. Thus, the demand for a technique with better functional outcomes made surgeons modify the straight anastomotic technique.

**Fig. 1:**
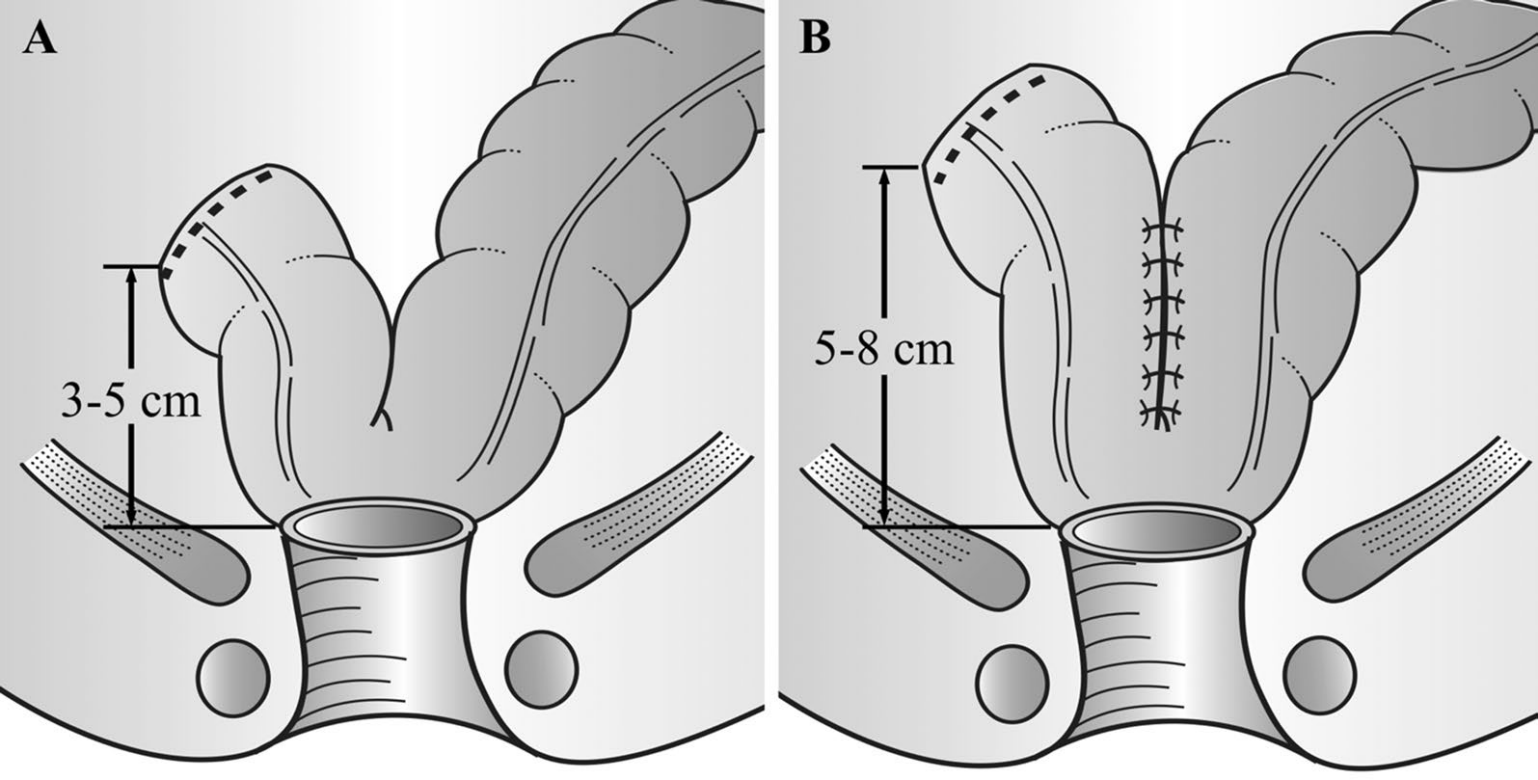
Side-to-end (**A**) and colonic J-pouch anastomosis (**B**)

Colonic J-pouch anastomosis was introduced by Lazorthes et al. [7] and Parc et al. [8] in 1986. A 5–8 cm-long colonic segment was considered as the optimum size of the J-pouch (Fig. 1B) [9]. Previous clinical trials and meta-analyses have proved that J-pouch could provide a better quality of life (QoL) and bowel functional outcomes compared with straight anastomosis [10, 11]. In some patients with the narrow pelvis or bulky mesentery, however, it is unable to perform colonic J-pouch [12]. Thus, another modified anastomotic technique, side-to-end anastomosis, which has been used since 1966, has gained attention [13]. Side-to-end anastomosis usually needs a 3–5 cm-long colonic segment (Fig. 1A). Multiple studies on the literature have shown that compared with straight anastomosis, side-to-end anastomosis has advantages in bowel functional and operative outcomes [14].

Previous trials [15–18] and meta-analyses [11, 19, 20] including a Cochrane review [11] have shown that J-pouch and side-to-end anastomosis had similar functional outcomes. Recent trials, however, reported that colonic J-pouch could provide a better short-term quality of life [21], but it may increase the anastomotic leakage rate [22]. Up to now, there is no clear evidence on which one of the two anastomotic techniques is better. Therefore, we conducted this meta-analysis to compare surgical and bowel functional outcomes and QoL in patients undergoing resection of a mid to upper rectal cancer with reconstruction using either a colonic J-pouch or a side-to-end pouch.

## Methods

This study was performed according to the recommendations of the preferred reporting items for systematic reviews and meta-analyses statement (PRISMA) [23]. In this study, colonic J-pouch was performed with 4–8 cm-long colonic segment, while side-to-end anastomosis was performed with 3–6 cm-long colonic segment.

### Study selection

A systematic literature search was performed in PubMed, Embase and Cochrane. Our search strategy included terms “rectal cancer, rectal cancers, colorectal cancer, colorectal cancers, rectal carcinoma, rectal carcinomas, colorectal carcinoma, or colorectal carcinomas” and “side-to-end, side to end, end to side, or end-to-side” and “J pouch, J-pouch, colonic-J-pouch, or coloanal-J-pouch”. The search details of each electronic database are shown in Supplementary table 1. The last search was performed on March 28, 2021. Furthermore, we also performed a manual search of references of articles and reviews to enrolled additional potentially eligible studies. All randomized controlled trials (RCTs) comparing colonic J-pouch with side-to-end anastomosis for rectal cancer were enrolled. Non-randomized trials, such as retrospective studies, reviews, meta-analyses, and comments, were excluded. If studies were reported on the same population of patients, the results were either combined, or the study with more detailed data was used.

### Data extraction

To avoid any mistakes or omissions, two authors reviewed all identified studies and extracted data using a standard paper-based extraction sheet independently. Whenever there were disagreements, a third reviewer was needed. The following items were extracted from each study: first author’s name, year of publication, the sample size of each arm, gender (male sex), age, the distance of the distal edge of the tumor from the anal verge, the distance of the anastomosis from the anal verge, neoadjuvant/adjuvant therapy, and outcomes of interest.

### Outcomes of interest

The main outcomes were bowel functional outcomes and QoL. Bowel functional outcomes mainly included three indexes: stool frequency, urgency, incomplete defecation and incontinence. According to the previous Cochrane meta-analysis [11], we recorded bowel functional outcomes and QoL at three time periods: short term (< 8 months), medium term (8–18 months), and long term (> 18 months). The secondary outcomes were surgical outcomes including operative time, postoperative hospital stay, postoperative complications, reoperation, and mortality.

### Quality assessment

We used the modified Jadad score system to assess the methodological quality of the randomized controlled trials (total score, 5; 1–2, low quality; 3–5, high quality) [24].

### Statistical analysis

We used Review Manager version 5.3 (The Cochrane Collaboration, Software Update, Oxford) for data analyses. *P* value < 0.05 was considered statistically significant. Continuous outcomes were analyzed using weighted mean difference (WMD), while dichotomous outcomes were analyzed using odds ratios (OR) or risk ratios (RR). If means and standard deviations (SDs) of continuous outcomes were not provided, we used methods described by Hozo et al. [25] to calculate means and SDs from means and range values or medians and range values. Besides, we used the Chi-squared test and Higgins I-squared test to calculate Heterogeneity. The value of *P* < 0.05 and I^2^ > 50% was considered as high heterogeneity, and therefore, a random-effects model was applied; otherwise, a fixed-effects model was applied. If high heterogeneity existed, we conducted sensitivity analysis by removing one study each time to decrease heterogeneity. Publication bias was assessed using Begg’s funnel plot.

## Results

**Fig. 2:**
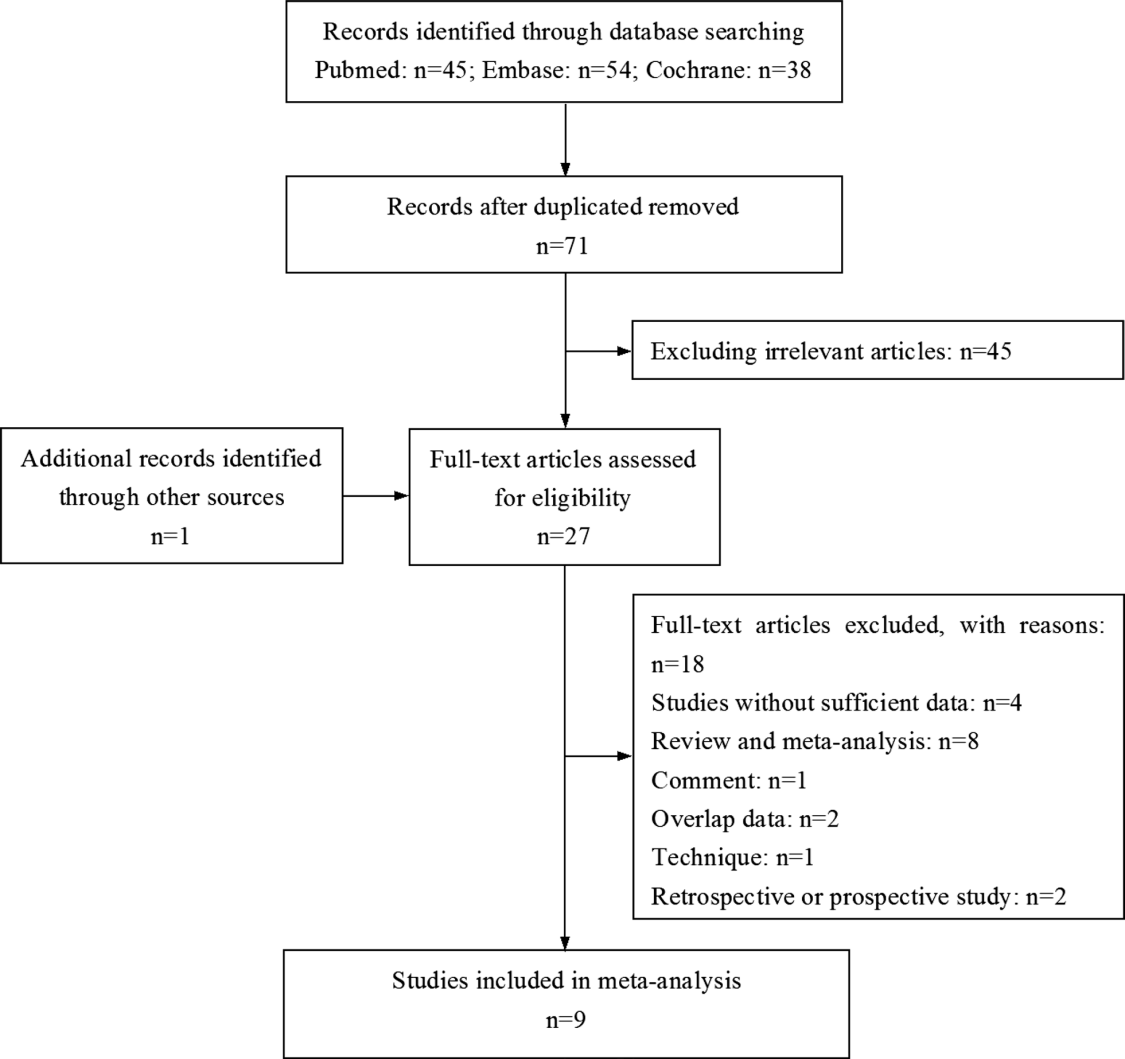
PRISMA diagram

The flow chart of the literature search is shown in Fig. 2. After duplicate data were removed, there were 71 records. After the initial review, 45 studies were excluded. Besides, one additional article was enrolled through a manual search. Finally, a total of 27 relevant studies were further evaluated. Of those studies, 18 reports were excluded due to the following reasons: four studies did not present sufficient data, eight studies were reviews or meta-analyses, one study was a comment, two studies had overlap patients with similar results, one study reported surgical technique, and two studies were retrospective or prospective study. Of the remaining nine articles, two papers by Machado et al. [16, 18] reported different outcomes which were based on the same trial, as well as another two papers by Marti et al. [26] and Ribi et al. [21]. Thus, nine articles incorporating 7 trials were enrolled in this meta-analysis [15–18, 21, 22, 26–28]. A total of 696 patients (330 by colonic J-pouch and 366 by side-to-end) were included.

### Patient demographics

The characteristics of the included studies were summarized in Table 1. No significant differences were found between colonic J-pouch and side-to-end groups in terms of the male sex, the distance of the distal edge of the tumor from the anal verge, the distance of the anastomosis from the anal verge, or neoadjuvant/adjuvant therapy. Although the age of J-pouch patients was younger than that of side-to-end, the mean age difference was only 1.60 [WMD = − 1.60, 95% CI (− 2.86, 0.35), I^2^ = 0%, *P* = 0.01].

**Table 1:** Study characteristics

Authors	No. of center	No. of patients		Male		Age		Tumor from AV	
		JP	SE	JP	SE	JP	SE	JP	SE
Doeksen	7	55	52	36	37	61.8/12.3	63.8/8.8	NR	NR
Huber	1	29	30	13	12	62.3	61.9	5.2/1.6	5.8/1.5
Jiang	1	24	24	12	15	62.3/3.3	64.9/2.8	7.9/1.5	8.6/0.3
Machado	1	50	50	27	32	63.8/11.3	65.0/11.8	9.5/3.0	9.8/2.8
Marti	15	63	95	38	62	63.4/13.7	63.9/9.5	6.0/2.5	7.5/2.3
Okkabaz	1	29	28	18	19	58.9/13.7	59.1/11.9	7.9/3.8	6.2/3.8
Parc	7	80	87	59	52	60.2/9.7	59.6/10.6	2.5/0.7	2.0/0.7

Authors	Anastomosis from AV		Neoadjuvant therapy		Adjuvant therapy		Quality of life assessment scale
	JP	SE	JP	SE	JP	SE	
Doeksen	NR	NR	55	52	0	0	COREFO, EORTC-QLQ-CR38, SF-36
Huber	3.8/0.8	4.2/0.5	NR	NR	NR	NR	NA
Jiang	4.8/0.2	5.3/0.3	12	10	2	1	NA
Machado	4.0/1.0	3.8/0.8	39	39	4	2	NA
Marti	NR	NR	49	72	34	53	FACT-C
Okkabaz	NR	NR	17	19	NR	NR	SF-36, SHIM, FISI, FSFI, OBVF
Parc	NR	NR	42	50	NR	NR	SF-12, FACT-C, IIEF, FISI

*JP* J-pouch, *SE* side-to-end, *AV* anal verge, *NR* not report, *NA* not applicable, *COREFO* COloREctal Functional Outcome, *FACT-C* Functional Assessment of Cancer Therapy-Colorectal, *SHIM* Sexual Health Inventory for Men, *FISI* Fecal Incontinence Severity Index, *FSFI* Female Sexual Function Index, *OBVF* Overactive Bladder-Validated Form, *IIEF* International Index of Erectile Function

### Quality of included studies

Although there were nine articles enrolled, only seven trials were involved. Thus, we just assessed those seven trials through seven articles. Of those seven trials, six trials had high quality, and one trial had low quality according to the modified Jadad score system (Table 2).

**Table 2:** Evaluation of methodological qualities of included randomized controlled trials

Items/author	Huber	Jiang	Okkabaz	Parc	Doeksen	Machado	Marti
Described as randomized	1	1	1	1	1	1	1
Appropriate randomization method described	1	1	1	1	1	1	1
Subject blinded to intervention	0	0	1	0	0	0	0
Evaluator blinded to intervention	0	0	0	0	0	0	0
Description of withdrawals and dropouts	0	1	1	1	1	1	1
Score	2	3	4	3	3	3	3

Methodological qualities of included randomized controlled trials were assessed by modified Jadad score system. Total score, 5; 1–2, low quality trial; 3–5, high quality trial

### Bowel functional outcomes

The bowel functional outcomes were shown in Table 3.

**Table 3:** Bowel functional outcomes

Bowel functional outcomes	No. of studies	J-pouch		Side-to-end		I^2^ (%)	Analysis model	OR/WMD	95% CI	P value
		Patients	Mean/rate	Patients	Mean/rate					
Baseline										
Stool frequency	3	122	1.1^#^	134	1.3^#^	71	Random	− 0.19^†^	− 0.42, 0.03	0.09
Urgency	3	92	11%^^^	97	8%^^^	19	Fixed	1.32^*^	0.52, 3.39	0.56
Incomplete defecation	2	74	4%^^^	74	1%^^^	0	Fixed	2.39^*^	0.34, 16.67	0.38
Short term										
Stool frequency	5	195	2.7^#^	208	3.0^#^	95	Random	− 0.30^†^	− 1.04, 0.44	0.42
Urgency	4	115	30%^^^	121	41%^^^	0	Fixed	0.53^*^	0.28, 1.00	0.05
Incomplete defecation	3	97	35%^^^	98	27%^^^	0	Fixed	1.65^*^	0.83, 3.28	0.15
Medium term										
Stool frequency	4	164	2.6^#^	173	2.9^#^	96	Random	− 0.32^†^	− 1.10, 0.45	0.41
Urgency	3	84	37%^^^	86	48%^^^	42	Fixed	0.67^*^	0.34, 1.29	0.23
Incomplete defecation	2	66	36%^^^	63	51%^^^	31	Fixed	0.56^*^	0.28, 1.12	0.10
Long term										
Stool frequency	3	135	1.4^#^	138	1.8^#^	91	Random	− 0.34^†^	− 0.84, 0.16	0.19
Urgency	2	55	22%^^^	51	39%^^^	0	Fixed	0.40^*^	0.16, 1.01	0.05
Incomplete defecation	2	55	33%^^^	51	39%^^^	0	Fixed	0.71^*^	0.31, 1.62	0.41

*OR* odds ratio, ^#^pooled mean, ^^^pooled rate, *WMD* weighted mean difference, *CI* confidence interval, *OR, ^†^WMD

#### Baseline

Three trials comparing J-pouch with side-to-end reported baseline stool frequency [17, 22, 28] and urgency [17, 18, 22]. The pooled data showed that the baseline stool frequency (J-pouch 1.1, side-to-end 1.3) had a random effects model of WMD = − 0.19, 95% CI (− 0.42, 0.03), I^2^ = 95%, P = 0.09 and urgency defined as yes or no (J-pouch 11%, side-to-end 8%) had a fixed effects model of OR = 1.32, 95% CI (0.52, 3.39), I^2^ = 19%, P = 0.56 which were comparable between the two groups. Only two trials reported baseline incomplete defecation defined as yes or no [17, 18], which was also similar in two groups [J-pouch 4%, side-to-end 1%, fixed effects model, OR = 2.39, 95% CI (0.34, 16.67), I^2^ = 0%, *P* = 0.38].

#### Short term

Five trials reported stool frequency [17, 18, 22, 27, 28], which was comparable between the two groups [J-pouch 2.7, side-to-end 3.0, random effects model, WMD = − 0.30, 95% CI (− 1.04, 0.44), I^2^ = 95%, *P* = 0.42]. The sensitivity analysis could not reduce the high heterogeneity. Four trials reported urgency (yes or no) [17, 18, 22, 27]. No significant difference was found between two groups [J-pouch 30%, side-to-end 41%, fixed effects model, OR = 0.53, 95% CI (0.28, 1.00), I^2^ = 0%, *P* = 0.05]. Three trials reported incomplete defecation (yes or no) [17, 18, 27], which was also comparable in two groups [J-pouch 35%, side-to-end 27%, fixed effects model, OR = 1.65, 95% CI (0.83, 3.28), I^2^ = 0%, *P* = 0.15].

#### Medium term

Four trials reported stool frequency [17, 18, 22, 28], which was comparable between the two groups [J-pouch 2.6, side-to-end 2.9, random effects model, WMD = − 0.32, 95% CI (− 1.10, 0.45), I^2^ = 96%, *P* = 0.41]. The sensitivity analysis could not reduce the high heterogeneity. Three trials reported urgency (yes or no) [17, 18, 22]. The pooled data showed no significant difference between two groups [J-pouch 37%, side-to-end 48%, fixed effects model, OR = 0.67, 95% CI (0.34, 1.29), I^2^ = 42%, *P* = 0.23]. Two trials reported incomplete defecation (yes or no) [17, 18], which was also comparable in two groups [J-pouch 36%, side-to-end 51%, fixed effects model, OR = 0.56, 95% CI (0.28, 1.12), I^2^ = 31%, *P* = 0.10].

#### Long term

Three trials reported stool frequency [16, 17, 28], which was comparable between the two groups [J-pouch 1.4, side-to-end 1.8, random effects model, WMD = − 0.34, 95% CI (− 0.84, 0.16), I^2^ = 91%, *P* = 0.19]. The sensitivity analysis could not reduce the high heterogeneity. Two trials reported urgency (yes or no) [16, 17]. The pooled data showed no significant difference between two groups [J-pouch 22%, side-to-end 39%, fixed effects model, OR = 0.40, 95% CI (0.16, 1.01), I^2^ = 0%, *P* = 0.05]. Two trials reported incomplete defecation (yes or no) [16, 17], which was also comparable in two groups [J-pouch 33%, side-to-end 39%, fixed effects model, OR = 0.71, 95% CI (0.31, 1.62), I^2^ = 0%, *P* = 0.41].

### Quality of life

Different trials used different questionnaires to assess QoL (Table 1). Only two trials [15, 22] which both used SF-36 questionnaire could be pooled together and these two trials involving 164 patients (84 by colonic J-pouch and 80 by side-to-end) in total served as our evaluation of QoL in this study. The SF-36 outcomes were shown in Table 4. At the baseline, the physical function and social function were comparable between J-pouch and side-to-end groups; however, the general health perception was slightly better in J-pouch [fixed effects model, WMD = 7.44, 95% CI (0.39, 14.48), I^2^ = 0%, *P* = 0.04]. At short term, the physical function [random effects model, WMD = 6.57, 95% CI (− 14.96, 28.10), I^2^ = 82%, *P* = 0.55], social function [random effects model, WMD = 5.20, 95% CI (− 12.47, 22.88), I^2^ = 67%, *P* = 0.56], and general health perception [random effects model, WMD = -0.48, 95% CI (− 19.39, 18.43), I^2^ = 79%, *P* = 0.96] were all similar in two groups. At medium term, there were no significant difference between J-pouch and side-to-end groups in terms of physical function [random effects model, WMD = 6.55, 95% CI (− 10.59, 23.69), I^2^ = 66%, *P* = 0.45], social function [random effects model, WMD = 0.18, 95% CI (− 14.64, 15.00), I^2^ = 70%, *P* = 0.98], and general health perception [random effects model, WMD = 0.94, 95% CI (− 12.62, 14.51), I^2^ = 72%, *P* = 0.89]. The sensitivity analysis could not reduce the high heterogeneity.

**Table 4:** Quality of life (SF-36)

Quality of life (SF-36)	No. of studies	J-pouch		Side-to-end		I^2^ (%)	Analysis model	WMD	95% CI	P value
		Patients	Mean	Patients	Mean					
Baseline										
Physical function	2	63	81.09	64	75.75	0	Fixed	5.34	− 1.61, 12.28	0.13
Social function	2	66	74.76	66	70.93	0	Fixed	3.83	− 4.56, 12.21	0.37
General health perception	2	65	67.63	65	60.19	0	Fixed	7.44	0.39, 14.48	0.04
Short term										
Physical function	2	39	75.85	46	69.28	82	Random	6.57	− 14.96, 28.10	0.55
Social function	2	40	68.91	46	63.71	67	Random	5.20	− 12.47, 22.88	0.56
General health perception	2	39	70.08	47	70.56	79	Random	− 0.48	− 19.39, 18.43	0.96
Medium term										
Physical function	2	46	84.13	44	77.58	66	Random	6.55	− 10.59, 23.69	0.45
Social function	2	48	82.94	45	82.76	70	Random	0.18	− 14.64, 15.00	0.98
General health perception	2	48	71.75	45	70.81	72	Random	0.94	− 12.62, 14.51	0.89

*WMD* weighted mean difference, *CI* confidence interval

### Surgical outcomes

**Fig. 3:**
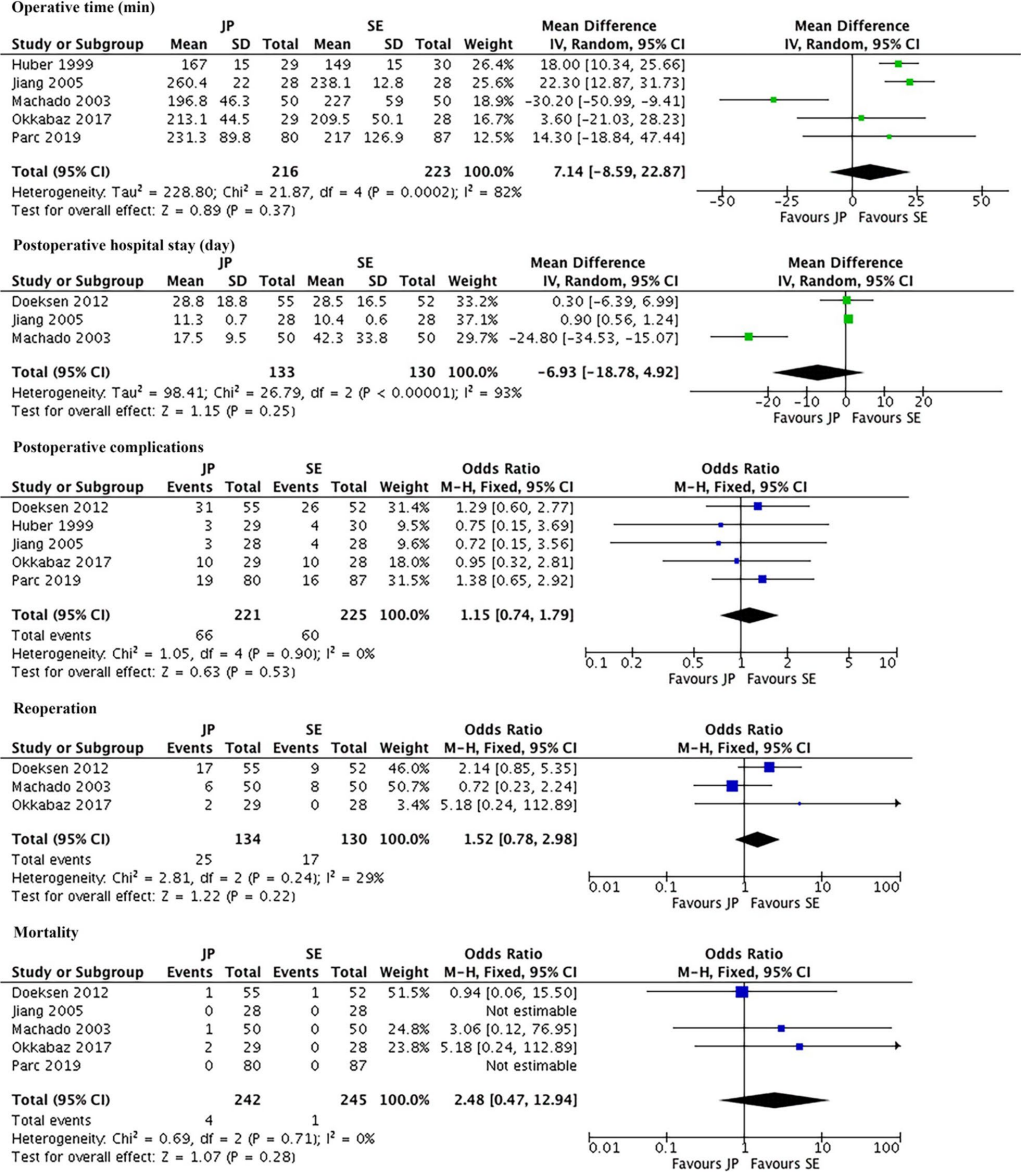
Meta-analysis of perioperative outcomes. *JP* J-pouch, *SE* side-to-end

**Fig. 4:**
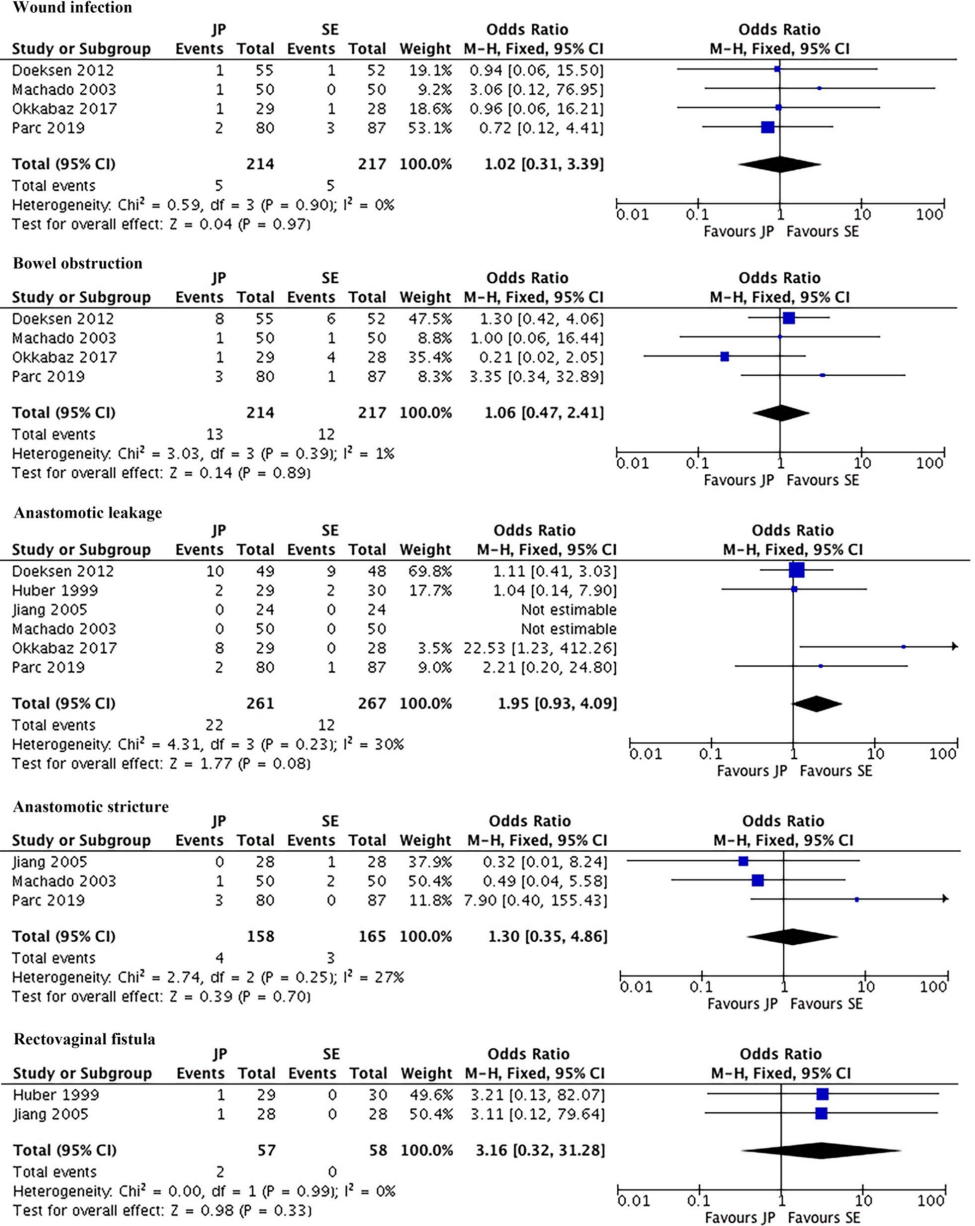
Meta-analysis of specific complications. *JP* J-pouch, *SE* side-to-end

Figures 3, 4 showed surgical outcomes. The pooled mean operative time was 216 min in J-pouch group and 209 min in side-to-end group. The pooled mean postoperative hospital stay was 20.9 days in J-pouch group and 27.8 days in side-to-end group. There was no statistically significance between the two groups in terms of operative time [random effects model, WMD = 7.14, 95% CI (− 8.59, 22.87), I^2^ = 82%, *P* = 0.37] and postoperative hospital stay [random effects model, WMD = − 6.93, 95% CI (− 18.78, 4.92), I^2^ = 93%, *P* = 0.25]. The sensitivity analysis could not reduce the high heterogeneity. The pooled postoperative complication rate, reoperation rate, and mortality rate were 29.9%, 18.7%, and 1.7% in J-pouch group and 26.7%, 13.1%, and 0.4% in side-to-end group, respectively. The postoperative complications [fixed effects model, OR = 1.15, 95% CI (0.74, 1.79), I^2^ = 0%, *P* = 0.53], reoperation [fixed effects model, OR = 1.52, 95% CI (0.78, 2.98), I^2^ = 29%, *P* = 0.22] and mortality [fixed effects model, RR = 2.48, 95% CI (0.48, 12.21), I^2^ = 0%, *P* = 0.28] were all comparable between two groups (Fig. 3). As for specific complications, pooled data showed that wound infection [fixed effects model, OR = 1.02, 95% CI (0.31, 3.39), I^2^ = 0%, *P* = 0.97], bowel obstruction [fixed effects model, OR = 1.06, 95% CI (0.47, 2.41), I^2^ = 1%, *P* = 0.89], anastomotic leakage [fixed effects model, OR = 1.95, 95% CI (0.93, 4.09), I^2^ = 30%, *P* = 0.08], anastomotic stricture [fixed effects model, OR = 1.30, 95% CI (0.35, 4.86), I^2^ = 27%, *P* = 0.70], and rectovaginal fistula [fixed effects model, OR = 3.16, 95% CI (0.32, 31.28), I^2^ = 0%, *P* = 0.33] were similar in two groups (Fig. 4).

### Publication bias

**Fig. 5:**
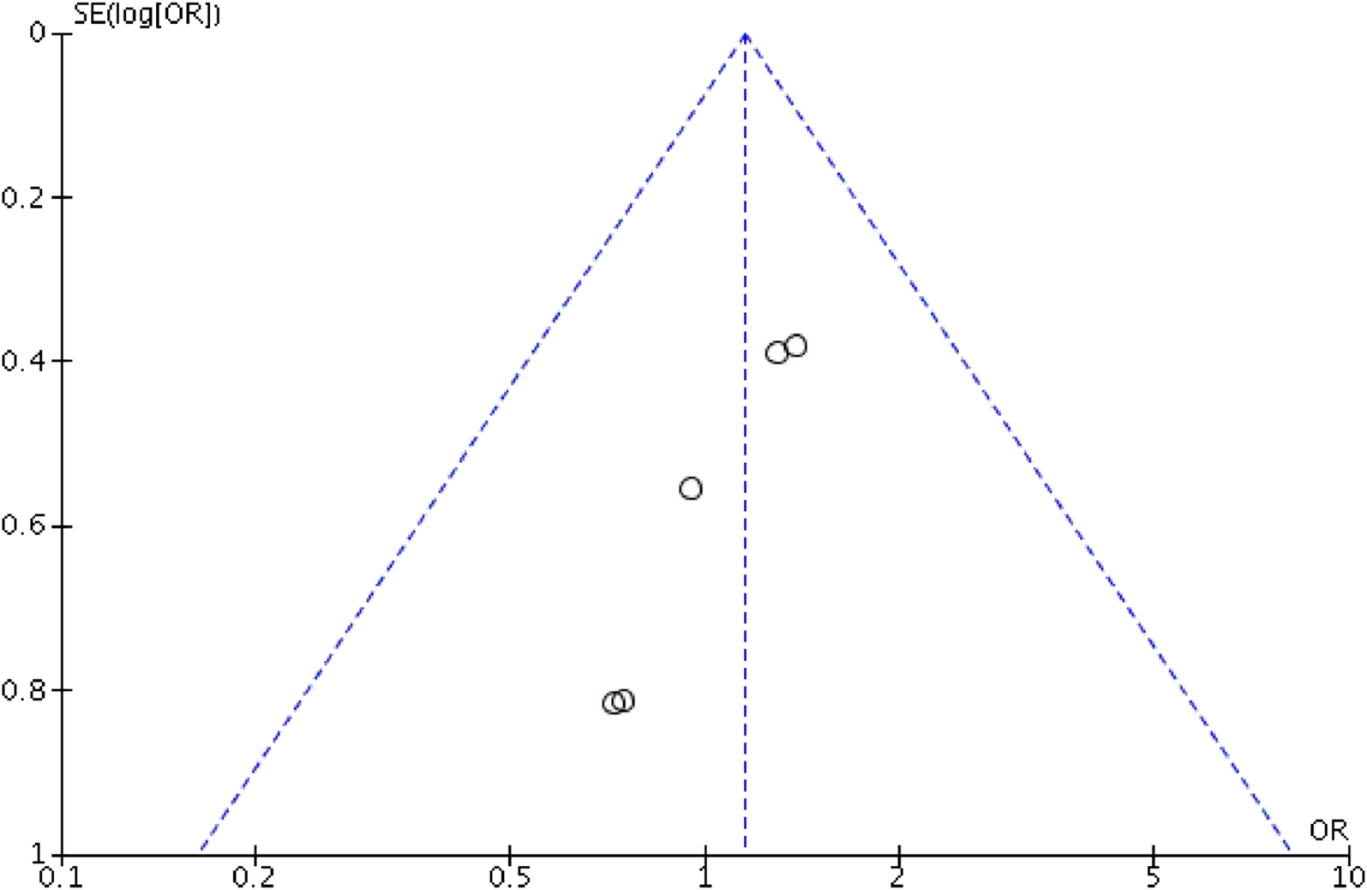
Funnel plots of the studies reporting on postoperative complications

Publication bias was assessed based on postoperative complications (Fig. 5). There was no obvious publication bias among the studies according to the Begg’s funnel plot.

## Discussion

With the advancement of surgical technique, the local recurrence rate after rectal cancer surgery has been decreased from 25–50% to 3–8% [29–32]. Naturally, it is time to focus on how to improve bowel functional outcomes and QoL for rectal cancer patients. One of the approaches is to modify the reconstructive technique. Though the most widely used reconstructive technique is the straight coloanal anastomosis, two modified reconstructive techniques, colonic J-pouch and side-to-end, can do provide better QoL and bowel functional outcomes compared with straight anastomosis [10, 11, 14]. Although the length of the colonic segment used for anastomosis was different in varied regions, the colonic J-pouch anastomosis usually needs a 5–8 cm-long colonic segment to build the J-pouch, while the side-to-end anastomosis needs a 3–5 cm-long colonic segment (Fig. 1). At present, there is no clear evidence on which one of the two modified anastomotic techniques is better. This meta-analysis enrolling nine articles incorporating 7 trials with a total of 696 patients (330 by J-pouch and 366 by side-to-end) showed that J-pouch was comparable with side-to-end anastomosis in terms of bowel functional outcomes, QoL (SF-36), and surgical outcomes.

Bowel dysfunction is one of the main concerns after sphincter-saving TME surgery. Huber et al. have reported that stool frequency was lower at 6 month after surgery in the J-pouch group [27], however, another trial by Jiang et al. reported it was higher at 6 month in the J-pouch group [17]. Our meta-analysis using only RCTs showed that there was no difference between two groups in terms of stool frequency at all three time-periods, as well as urgency, or incomplete defecation, which were supported by previous meta-analysis [19, 20, 33]. Six studies reported the incontinence outcome, however, the measurement method and/or the data type of incontinence was different [15–18, 26, 28], which we could not pool together. The meta-analysis by Siddiqui et al. have analyzed the pressure and volumetric outcomes and documented that those results were comparable in J-pouch and side-to-end groups [20]. After that, no recent trial reported pressure or volumetric outcomes. Therefore, we did not analyze those two outcomes.

In relation to QoL, different trials used different questionnaires, including COloREctal Functional Outcome (COREFO) [15], EORTC-QLQ-CR38 [15], SF-36 [15, 22], Functional Assessment of Cancer Therapy-Colorectal (FACT-C) [26, 28], Sexual Health Inventory for Men (SHIM) [22], Fecal Incontinence Severity Index (FISI) [22, 28], Female Sexual Function Index (FSFI) [22], Overactive Bladder-Validated Form (OBVF) [22], SF-12 [28], and International Index of Erectile Function (IIEF) [28]. According to data provided by those trials, we could only analyze the SF-36 outcomes (physical function, social function and general health perception), and the pooled data showed no significant difference was observed at short or medium term between two groups. Previous literatures documented that bowel dysfunction can influence QoL after surgery for rectal cancer [34–36]. As this meta-analysis showed no significant difference existed between J-pouch and side-to-end in terms of bowel functional outcomes, it was not surprising that physical function, social function and general health perception were comparable in two groups.

Theoretically, J-pouch anastomosis is technically more demanding, the formation of which should take more time. However, the operative time was comparable in J-pouch and side-to-end groups, which was also supported by previous meta-analysis [20]. The possible reason is that the J-pouch is performed using a stapler, which only takes very little time. No significant difference was observed in the postoperative hospital stay. The postoperative complication rates of enrolled studies were 10.3–56.4% in the J-pouch group and 13.3–50.0% in the side-to-end group. The big difference in complication rates was due to the different diagnostic criteria in different departments. The pooled postoperative complication rate was 29.9% and 26.7% in J-pouch and side-to-end groups, respectively, which was similar in the two groups. As for anastomotic leakage rate, previous literatures have reported that the incidence of anastomotic leakage ranges from 1 to 21% in patients undergoing a coloanal anastomosis [37–41]. In our meta-analysis, the anastomotic leakage rate was comparable in two groups (8.4% in J-pouch and 4.5% in side-to-end, P = 0.08), which was also supported by previous meta-analysis [19]. Other complications (wound infection, bowel obstruction, anastomotic stricture, and rectovaginal fistula) were comparable between J-pouch and side-to-end groups.

Two meta-analyses have shown that J-pouch and side-to-end anastomosis had similar functional outcomes [19, 33]; after that, another three RCTs comparing colonic J-pouch and side-to-end have published recently with controversial results [21, 22, 26, 28]. Therefore, we conducted this update meta-analysis. Although the current analysis still included a relatively small amount of RCTs as well as a small number of patients, it provided valuable data for comparing the colonic J-pouch and side-to-end anastomosis after sphincter-saving TME surgery, considering that there was no large cohort study (more than 100 patients in each group). However, this meta-analysis has some limitations need to be highlighted. First of all, high heterogeneity existed in some analyses, and the sensitivity analysis could not reduce those heterogeneities. The heterogeneity might be influenced by some factors, such as the patient’s sex, the experience of the surgeon, the surgical technique (open/laparoscopic), and (neo) adjuvant therapy. Second, there were some completed trials in clinical trial registration, however, the results of those trials were not published, which could induce some bias. Third, different trials used different QoL questionnaires due to the lack of good QoL parameters, we could only analyze the SF-36 outcomes in 2 studies. Fourth, none of these nine studies reported the outcomes separately for men and women; thus, we could not analyze this outcome. Fifth, we did not analyze the nighttime incontinence because no studies reported this outcome. We hope that future studies would address these issues.

## Conclusions

The currently limited evidence suggests that colonic J-pouch and side-to-end anastomosis are comparable in terms of bowel functional outcomes, QoL, and perioperative outcomes. Hence, surgeons may choose either of the two techniques for anastomosis. A large sample randomized controlled study comparing colonic J-pouch and side-to-end anastomosis for rectal cancer is warranted.

## Data Availability

The dataset used and/or analyzed during the current study are available from the corresponding author on reasonable request.

## Abbreviations

ARS: Anterior resection syndrome
OR: Odds ratios
PRISMA: Preferred reporting items for systematic reviews and meta-analyses statement
QoL: Quality of life
RR: Risk ratios
SD: Standard deviation
TME: Total mesorectal excision
WMD: Weighted mean difference
RCTs: Randomized controlled trials
